# Effect of human, bovine and ovine prolactin on DNA synthesis by organ cultures of benign human breast tumours.

**DOI:** 10.1038/bjc.1979.278

**Published:** 1979-12

**Authors:** C. W. Welsch, S. E. Dombroske, M. J. McManus, G. Calaf

## Abstract

Ten benign breast tumours from 9 female patients (8 with fibrocystic disease and 1 with fibroadenoma) and 1 male patient (with gynaecomastia) were processed into slices and individually cultured for 2 days in serum-free Medium 199. [3H]-TdR was added to the culture medium to assess DNA synthesis. The addition of human prolactin to the culture medium (500 ng/ml) significantly (0.05 greater than P greater than 0.01) increased DNA synthesis; all 9 biopsy specimens from the 9 female patients responded positively to this hormone. Ovine prolactin (500 ng/ml) and bovine prolactin (500 ng/ml) increased the mean incorporation of [3H]-TdR into extracted DNA and increased the mean number of [3H]-TdR-labelled cells, but this increase did not reach the 5% level of probability. The sole case of male breast dysplasia analysed in this study did not respond to either human, ovine or bovine prolactin. These results provide evidence that human prolactin and, to a lesser degree, ovine and bovine prolactin are direct mitogenic stimulants to the epithelium in human (female) benign breast tumours.


*B. J. Cancer* (1979) **40**, 866

### EFFECT OF HUMAN, BOVINE AND OVINE PROLACTIN ON DNA SYNTHESIS BY ORGAN CULTURES OF BENIGN HUMAN BREAST TUMOURS

C. W. WELSCH, S. E. DOMBROSKE, M. J. McMANUS AND G. CALAF

*From the Department of Anatomy, Michigan State University, East Lansing, Michigan, 48824, U.S.A. and the Department of Biology, Faculty of Science, University of Chile, Santiago, Chile*

Received 15 June 1979 Accepted 7 August 1979

**Summary.—Ten benign breast tumours from 9 female patients (8 with fibrocystic disease and 1 with fibroadenoma) and 1 male patient (with gynaecomastia) were processed into slices and individually cultured for 2 days in serum-free Medium 199. [$^3$H]-TdR was added to the culture medium to assess DNA synthesis. The addition of human prolactin to the culture medium (500 ng/ml) significantly ($0{\cdot}05 > P > 0{\cdot}01$) increased DNA synthesis; all 9 biopsy specimens from the 9 female patients responded positively to this hormone. Ovine prolactin (500 ng/ml) and bovine prolactin (500 ng/ml) increased the mean incorporation of [$^3$H]-TdR into extracted DNA and increased the mean number of [$^3$H]-TdR-labelled cells, but this increase did not reach the 5% level of probability. The sole case of male breast dysplasia analysed in this study did not respond to either human, ovine or bovine prolactin. These results provide evidence that human prolactin and, to a lesser degree, ovine and bovine prolactin are direct mitogenic stimulants to the epithelium in human (female) benign breast tumours.**

IT HAS BEEN REPORTED from numerous laboratories that a hyperprolactinaemia in intact mice or rats results in a profound increase in the growth of the mammary gland (Bardin *et al.*, 1966; Welsch *et al.*, 1968). The mammotrophic effects of prolactin on the rodent mammary gland may be direct and/or indirect, *i.e. via* the ovary. To determine whether or not prolactin is *directly* mitogenic to the mammary gland, a number of laboratories have used cell or organ culture to resolve this question. The results of these studies have not been consistent or conclusive. In mice, Mayne & Barry (1970) and Oka & Topper (1972) found no stimulatory effect of prolactin on DNA synthesis by organ cultures of mammary gland, whereas Mukherjee *et al.* (1973) have provided evidence to the contrary. In rats, Dilley (1971) and Hallowes *et al.* (1973) have reported that prolactin is mitogenic *in vitro* to the mammary epithelium, whereas Koyama *et al.* (1972) did not see any stimulatory effect of prolactin on mammary-gland growth *in vitro*.

Explants of human breast, both normal and hyperplastic (unlike scirrhous carcinoma) can also be readily maintained in short-term organ culture (Welsch *et al.*, 1976; Welsch & McManus, 1977; Welsch *et al.*, 1978). Ceriani *et al.* (1972), using strictly morphological criteria, concluded that the addition of ovine prolactin to culture media containing explants of normal human breast tissues stimulated the epithelium contained in the explants. Flaxman & Lasfargues (1973), using objective criteria ([$^3$H]-TdR labelling index), reported that bovine prolactin was mitogenic to explants of normal human breast tissue *in vitro*. Dilley & Kister (1975)

Reprint requests to: C. W. Welsch, Department of Anatomy, Michigan State University, East Lansing, MI 48824, U.S.A.

reported that human prolactin but not ovine prolactin was capable of stimulating *in vitro* growth of normal human breast tissues. Although several laboratories have reported the effects of prolactin on the metabolic activity of human breast carcinoma *in vitro*, with varying conclusions (for review, see Welsch & Nagasawa, 1977), to our knowledge these 3 reports are the sole studies which have attempted to determine whether or not prolactin has a direct mitogenic effect on normal human breast epithelium *in vitro*.

Biopsy specimens of benign human breast tissues contain an array of normal and hyperplastic epithelial tissues. In recent reports, we have provided evidence that DNA synthesis by the epithelium of these benign dysplasias is stimulated *in vitro* (Welsch & McManus, 1977) and *in vivo* (McManus *et al.*, 1978; McManus & Welsch, 1979) by human placental lactogen (HPL) but not by human growth hormone (HGH) (Welsch *et al.*, 1978). The purpose of the work reported here was to ascertain *via* organ-culture experiments whether prolactin, obtained from 3 different species (human, bovine and ovine), is capable of stimulating DNA synthesis of the epithelium contained in these human breast dysplasias.

### MATERIALS AND METHODS

Ten benign breast tumours obtained from 9 female patients (8 with fibrocystic disease and 1 with fibroadenoma) and 1 male patient (with gynaecomastia) were placed in a chilled holding medium and returned to the laboratory within 30 min. The biopsy specimens were immediately and carefully trimmed of adipose tissue while immersed in the holding medium. All tissue preparations were performed in a laminar flow hood under aseptic conditions.

Slices of biopsy specimens were prepared with the aid of a Stadie-Riggs tissue slicer and a No. 10 Bard-Parker surgical blade. Each biopsy specimen provided 5–15 large slices 0·1–0·3 mm thick and ranging from 10 to 15 mm in diameter. Each slice was processed by a series of halvings with a surgical blade, each half being halved again and again until the slices measured 1 × 1 mm. These small slices were pooled and placed in 10 × 30mm Falcon disposable Petri dishes, 10 slices/dish. In addition, a single larger slice (3 × 3 mm) was added to each small Petri dish. Each Petri dish contained 2·0 ml of the culture medium.

Each biopsy specimen was divided into 4 groups (a control and 3 experimental groups). Each group had 9 small Petri dishes containing a total of 90 small slices and 9 larger slices. The small Petri dishes were placed in a covered water-saturated larger Falcon disposable Petri dish (15 × 100 mm), 3 small dishes per larger dish. These Petri dishes were then placed in a small gassing chamber and housed in an incubator at 37°C. The chambers were continuously infused with gas (95% $O_2$–5% $CO_2$) during the culture period. All biopsy specimens were individually cultured; slices from different specimens were never combined. The large number of randomly selected small slices per group provides reasonable assurance that an equal quantity of epithelium is distributed among the groups at the onset of culture.

The culture medium used in these studies was Medium 199, modified Earle's salts (1250 mg $NaHCO_3$/l) obtained from Grand Island Biological Co., Grand Island, NY. The hormones used in this study were human prolactin (NIH-hPr-VLS-3), bovine prolactin (NIH-B-3) and ovine prolactin (NIH-S-12) and were added to the culture media at a concentration of 500 ng/ml. After all additions, the media were passed through a Millipore filter (0·45 $\mu$m), added to the Petri dishes, and the entire culture assembly was frozen (−20°C) until the biopsy specimens were brought to the laboratory (within 1 month).

At the end of culture, 4 h before termination, sterile (methyl-$^3$H)-thymidine ([$^3$H]-TdR) (New England Nuclear, Boston, MA, 56·9 Ci/mmol) was added to the culture medium at a concentration of 1·0 $\mu$Ci/ml. Termination of the cultures was designed to facilitate quick removal of the small slices from the media in order to obtain a wet weight for each group, and then storage in 0·9% NaCl solution at −20°C until DNA extraction and analysis. The larger slices were also quickly removed, fixed in 10% buffered formalin, and stored for radioautographic and histological analyses.

For DNA extraction and analysis, the tissues from each group were ground in 0·9%

NaCl solution with a Willems Polytron homogenizer. An equal volume of 20% trichloroacetic acid was added to the homogenate; the resulting precipitate was centrifuged (6800 *g*) and washed twice with 10% trichloroacetic acid. The precipitate was then washed twice in sodium acetate–methanol solution and in chloroform–methanol, once in 100% ethanol, and once in 100% ethyl ether, in that order, to remove lipid and $H_2O$. In all the foregoing procedures, the preparations were kept constantly cold. The defatted-dehydrated extract was placed in a ventilated fume hood (12–18 h), then in a vacuum desiccator (24 h), and subsequently weighed.

The defatted-dehydrated extract was digested (3 h, 37°C) with repeated stirrings in 0·3N KOH. The preparation was cooled, precipitated with cold 10% perchloric acid, centrifuged (6800 *g*) and washed twice. The precipitate was then incubated for 30 min with constant sitrring in hot (70°C) 5% perchloric acid in which the DNA was soluble. This preparation was cooled, centrifuged (6800 *g*) and washed twice with cold 5% perchloric acid. The supernatant was collected for DNA and [$^3$H]-TdR analysis. DNA content was quantitatively determined (in duplicate) by the diphenylamine colorimetric method of Burton (1956). Calf thymus DNA (Sigma Chemical Co., St. Louis, MO) was used as a standard. The [$^3$H]-TdR content was determined by pipetting aliquots (in triplicate) of the supernatant into modified Bray's scintillation fluid. The samples were counted in a Beckman LC-100C liquid scintillation counter with a counting efficiency of 51%. The results were expressed as ct/min of [$^3$H]-TdR per $\mu$g DNA. Significance of differences between mean ct/min/$\mu$g DNA values of each group was analysed by the *t* test for paired observations.

For radioautographic analysis, the fixed slices (9 slices/group) were embedded in Parablast, a synthetic paraffin, sectioned at 5–7 $\mu$m and mounted on glass slides. Six sections were used for radioautography. Each slice, therefore, provided 6 sections. The 6 sections were taken at 20–50$\mu$m intervals through the entire slice. The slides for radioautography were dipped in Kodak NTB2 nuclear tract emulsion (Eastman Kodak Co., Rochester, NY), dried and exposed to the emulsion for 3 weeks at 4°C. They were then developed and stained (haematoxylin and eosin) by a standard method (Walker, 1959).

Radiolabelled epithelial cells, for a total of 6 sections for each slice, were counted. Only cells bearing >10 silver grains were scored as positive. With the use of a $\mu$m grid, an estimate of the area of the epithelium in the 6 sections of each slice was obtained and the number of radiolabelled cells per slice per unit area of epithelium was calculated. A unit area of epithelium is defined as 10 squares of the 100-square grid, which is equivalent to 0·1 mm$^2$. This method, previously published (McManus *et al.*, 1978) is time-conserving, precise and allows for an analysis of a larger area of tissue. The mean number of radiolabelled cells per unit epithelium and standard error of the mean were calculated. Mean differences between number of radiolabelled epithelial cells per slice per unit area of epithelium for each group were evaluated statistically by Student's *t* test for paired observations.

### RESULTS

The addition of human prolactin to 2-day organ cultures of 9 benign human breast tumours obtained from 9 female patients significantly ($P < 0·05$) increased the mean incorporation of [$^3$H]-TdR into chemically extracted DNA (Fig. 1) and significantly ($P < 0·01$) increased the mean number of [$^3$H]-TdR-labelled epithelial cells per unit area of epitehlium (Table). *All* 9 human breast-tumour biopsy specimens showed increases in the specific activity of [$^3$H]-TdR in DNA and increases in the number of [$^3$H]-TdR-labelled cells when human prolactin was added to the culture medium.

The addition of ovine or bovine prolactin to the culture medium increased the mean incorporation of [$^3$H]-TdR into chemically extracted DNA (Fig.) and increased the mean number of [$^3$H]-TdR-labelled epithelial cells (Table) but these increases did *not* reach the 5% level of statistical probability. Only 3/9 human breast tumour biopsy specimens showed increases in the specific activity of [$^3$H]-TdR in DNA and increases in the number of [$^3$H]-TdR-labelled cells when ovine or bovine prolactin was added to the culture media. The sole fibroadenoma analysed in this study did not respond significantly to

TABLE.—*Effect of human, ovine and bovine prolactin on number of [$^3H$]-TdR-labelled epithelial cells in 2-day organ cultures of 9 benign human breast tumours*

| Treatment | Mean labelling index (No. labelled cells/unit area of epithelium*) | Significance (controls) |
|---|---|---|
| Control | 36·0 ± 7·1 | *vs* |
| OvPrl | 46·1 ± 11·2 | N.S. |
| BovPrl | 52·6 ± 11·6 | N.S. |
| HuPrl | 49·3 ± 7·8 | $P < 0·01$ |

Mean ± s.e. of 9 individually cultured human breast tumours obtained from 9 female patients.

FIG.—Effect of human, ovine and bovine prolactin on [$^3$H]-TdR incorporation into DNA of 2-day organ cultures of 9 benign human breast tumours.

Means of 9 individually cultured human breast tumours obtained from 9 female patients are shown with the s.e. indicated.

Control *vs* HuPrl, $P < 0·05$.

Control *vs* OvPrl or BovPrl, no significant difference.

ovine or bovine prolactin but did respond to human prolactin. There were no readily apparent morphological changes due to prolactin treatment; *all* groups, including controls, showed excellent preservation of original morphology and cellular viability.

Radioautographic analysis indicates that virtually all of the [$^3$H]-TdR-labelled cells were epithelial; silver grains were rarely seen over the nuclei of fibroblasts.

Because of the rarity of human male breast tumours, we seldom have the opportunity to obtain and culture these breast dysplasias. Interestingly, in the sole male breast dysplasia analysed in this study (gynaecomastia) neither [$^3$H]-TdR incorporation into chemically extracted DNA or number of [$^3$H]-TdR-labelled cells were increased by human, ovine or bovine prolactin, although this specimen was adequately maintained in culture.

### DISCUSSION

The primary structure of human bovine and ovine prolactin has been studied by a number of laboratories (Lewis *et al.*, 1972; Wallis, 1974). All 3 hormones have a mol. wt of ~22,500 and have 3 disulphide bonds. Human and ovine prolactin have 198 amino-acid residues and bovine prolactin has 199. The amino-acid sequence homologies of ovine and bovine prolactin are nearly identical. Only 3 differences in amino-acid sequence have been observed: at Residue 108, alanine (Bov) replaces valine (Ov), at Residue 165, tyrosine (Bov) replaces histidine (Ov), and an extra residue of leucine at Position 88 in bovine prolactin. In contrast, the sequence homology of human prolactin, when compared with ovine or bovine prolactin, is similar but far from being identical. Human and bovine (or ovine) prolactin differ in sequence homologies at ~20% of the amino-acid residues. It is clear, therefore, that bovine and ovine prolactin are structurally nearly identical whereas human prolactin has significant structural deviation from the other two.

This study provides evidence that human prolactin is mitogenic to the epithelium of benign human breast tumours. When human prolactin was a component of the culture medium, *both* the specific activity of [$^3$H]-TdR in chemically extracted DNA and the number of [$^3$H]-TdR-labelled cells were increased in *all* 9 of the female biopsy specimens analysed. The 2 techniques of assessing DNA synthesis, therefore, were in total agreement. The radioautographic technique is important for it is not only quantitative but also qualitative, *i.e.* it allows one to determine which cell type (epithelial or connective tissue) is responding to the hormonal stimulus. It is clear from these results that it was the epithelial elements of the benign human breast tumours that responded to prolactin; rarely were silver grains seen over the connective-tissue elements of this tissue. Dilley & Kister (1975) briefly reported that human prolactin stimulated DNA synthesis of organ cultures of normal human breast tissues. Their results are similar to ours despite a slightly different experimental design. They cultured the explants for 4 days, used a different chemically defined culture medium, added insulin to the culture medium, and used a 10-fold greater medium concentration of human prolactin. The studies of Dilley & Kister (1975) and ourselves are the only ones, to our knowledge, attempting to determine whether *human* prolactin is mitogenic to the non-cancerous human breast.

Ovine (Ceriani *et al.*, 1972) and bovine (Flaxman & Lasfargues, 1973) prolactin have been reported previously to stimulate growth of organ cultures of normal human breast tissues. We were able to demonstrate a mean increase in [$^3$H]-TdR incorporation into DNA and a mean increase in the number of [$^3$H]-TdR-labelled cells when these pituitary peptides were added to the culture media, although this mean increase was *not* statistically significant ($P > 0{\cdot}05$). The response of the cultured human breast tissue to the herbivore prolactins, unlike that with human prolactin, was inconsistent. It is interesting to note that, of the 3 biopsy specimens responding to ovine prolactin and the 3 responding to bovine prolactin, 2 were from the same biopsy. This suggests that certain human breast specimens can respond to herbivore prolactins, but others cannot. Although the herbivore prolactins may be capable of stimulating growth of certain human breast tissues, it appears that these prolactins are less efficacious, certainly less consistent, than human prolactin in this respect. Similarly Kleinberg & Todd (1978) reported that human prolactin was considerably more effective than ovine prolactin in stimulating $\alpha$-lactalbumin synthesis by organ cultures of primate mammary tissues.

It is interesting to note that of the 10 biopsy specimens analysed in this study the only one not responding to human prolactin was the only one from a male patient (gynaecomastia). This specimen did not respond to ovine or bovine prolactin either. No difficulty was encountered in maintaining this specimen in culture, as cell viability, as with the specimens from female patients, was well maintained. Whilst one cannot draw definitive conclusions from data derived from a single specimen, this observation is worthy of note.

The tissue analysed in this study was derived from biopsy specimens of benign breast disease, most of which were histopathologically diagnosed as fibrocystic disease. All these specimens contain varying amounts of normal and hyperplastic breast tissues, the former component being predominant. (The term "normal" refers to tissue that appears normal morphologically.) In the previously mentioned studies of Dilley & Kister (1975), Ceriani *et al.* (1972) and Flaxman & Lasfargues (1973), the tissue was acquired either from benign breast biopsy specimens or from mastectomized carcinomatous breasts. The tissue obtained for culture, which was described as normal, was derived from sites distant from the primary dysplasia. The tissue which we analysed in our study was derived directly from biopsy of a breast with benign dysplasia. Because our tissue was derived from a *bona fide* benign tumour, we hesitate to call it normal, despite the fact that most of the specimens appear normal morphologically. The tissue which we analysed, however, may be similar to that analysed in the aforementioned studies.

The *in vitro* stimulation by prolactin of DNA synthesis by the epithelial tissue contained in benign human breast tumours suggests that this pituitary peptide may play a role in the aetiology of this disease. A recent report by Cole *et al.* (1977) has provided evidence that women with benign breast disease have higher than normal blood prolactin levels. Whilst conclusive evidence linking benign breast disease to prolactin is lacking, the results of our study, and the aforementioned studies, do provide evidence that such a link is possible.

This study was supported by American Cancer Society Research Grant No. BC-220-C.

### REFERENCES

BARDIN, C. W., LIEBELT, A. G. & LIEBELT, R. A. (1966) Mammary gland development after hypophyseal isografts in intact mice of high and low mammary cancer strains. *J. Natl Cancer Inst.*, **36**, 259.

BURTON, K. A. (1956) A study of the conditions and mechanism of the diphenylamine reaction for the colorimetric estimation of deoxyribonucleic acid. *Biochem. J.*, **62**, 315.

CERIANI, R. L., CONTESSO, G. P. & NATAF, B. M. (1972) Hormone requirement for growth and differentiation of the human mammary gland in organ culture. *Cancer Res.*, **32**, 2190.

COLE, E. N., SELWOOD, R. A., ENGLAND, P. C. & GRIFFITHS, K. (1977) Serum prolactin concentrations in benign breast disease throughout the menstrual cycle. *Eur. J. Cancer*, **13**, 597.

DILLEY, W. G. (1971) Morphogenic and mitogenic effects of prolactin on rat mammary gland *in vitro*. *Endocrinology*, **88**, 514.

DILLEY, W. G. & KISTER, S. J. (1975) *In vitro* stimulation of human breast tissue by human prolactin. *J. Natl Cancer Inst.*, **55**, 35.

FLAXMAN, B. A. & LASFARGUES, E. Y. (1973) Hormone-independent DNA synthesis by epithelial cells of adult human mammary gland in organ culture. *Proc. Soc. Exp. Biol. Med.*, **143**, 371.

HALLOWES, R. C., WANG, D. Y. & LEWIS, D. J. (1973) The lactogenic effects of prolactin and growth hormone on mammary gland explants from virgin and pregnant Sprague-Dawley rats. *J. Endocrinol.*, **57**, 253.

KLEINBERG, D. L. & TODD, J. (1978) α-Lactalbumin in human and subhuman primate normal mammary tissue and in human breast cancer as a marker for prolactin activity. *Cancer Res.*, **38**, 4318.

KOYAMA, H., SINHA, D. & DAO, T. L. (1972) Effects of hormones and 7,12-dimethylbenzanthracene on rat mammary tissue grown in organ culture. *J. Natl Cancer Inst.*, **48**, 1671.

LEWIS, U. J., SINGH, R. N. P. & SEAVEY, B. K. (1972) Problems in the purification of human prolactin. In *Prolactin and Carcinogenesis*, Eds. Boyns & Griffiths. Cardiff: Alpha Omega Alpha. p. 4.

MAYNE, R. & BARRY, J. M. (1970) Biochemical changes during development of mouse mammary tissue in organ culture. *J. Endocrinol.*, **46**, 61.

MCMANUS, M. J., DOMBROSKE, S. E., PIENKOWSKI, M. M. & 5 others (1978) Successful transplantation of human benign breast tumors into the athymic nude mouse and demonstration of enhanced DNA synthesis by human placental lactogen. *Cancer Res.*, **38**, 2343.

MCMANUS, M. J. & WELSCH, C. W. (1979) DNA synthesis of benign human breast tumors in the untreated athymic nude mouse: An *in vivo* model to study hormonal influences on growth of human breast tissues. *Cancer* (In press).

MUKHERJEE, A. S., WASHBURN, L. L. & BANERJEE, M. R. (1973) Role of insulin as a permissive hormone in mammary gland development. *Nature*, **246**, 159.

OKA, T. & TOPPER, Y. J. (1972) Is prolactin mitogenic for mammary epithelium? *Proc. Natl Acad. Sci.,*, U.S.A., **69**, 1693.

WALKER, B. E. (1959) Radioautographic observations on regeneration of transitional epithelium. *Tex. Rep. Biol. Med.*, **17**, 375.

WALLIS, M. (1974) The primary structure of bovine prolactin. *FEBS Lett.*, **44**, 205.

WELSCH, C. W., CALAF DE ITURRI, G. & BRENNAN, M. J. (1976) DNA synthesis of human, mouse and rat mammary carcinomas *in vitro*. *Cancer*, **38**, 1272.

WELSCH, C. W., CLEMENS, J. A. & MEITES, J. (1968) Effects of multiple pituitary homografts or progesterone on 7, 12-dimethylbenzanthracene-induced mammary tumors in rats. *J. Natl Cancer Inst.*, **41**, 465.

WELSCH, C. W., DOMBROSKE, S. E. & MCMANUS, M. J. (1978) Effects of insulin, human placental lactogen and human growth hormone on DNA synthesis in organ cultures of benign human breast tumours. *Br. J. Cancer*, **38**, 258.

WELSCH, C. W. & MCMANUS, M. J. (1977) Stimulation of DNA synthesis by human placental lactogen or insulin in organ cultures of benign human breast tumors. *Cancer Res.*, **37**, 2257.

WELSCH, C. W. & NAGASAWA, H. (1977) Prolactin and murine mammary tumorigenesis: A review. *Cancer Res.*, **37**, 951.


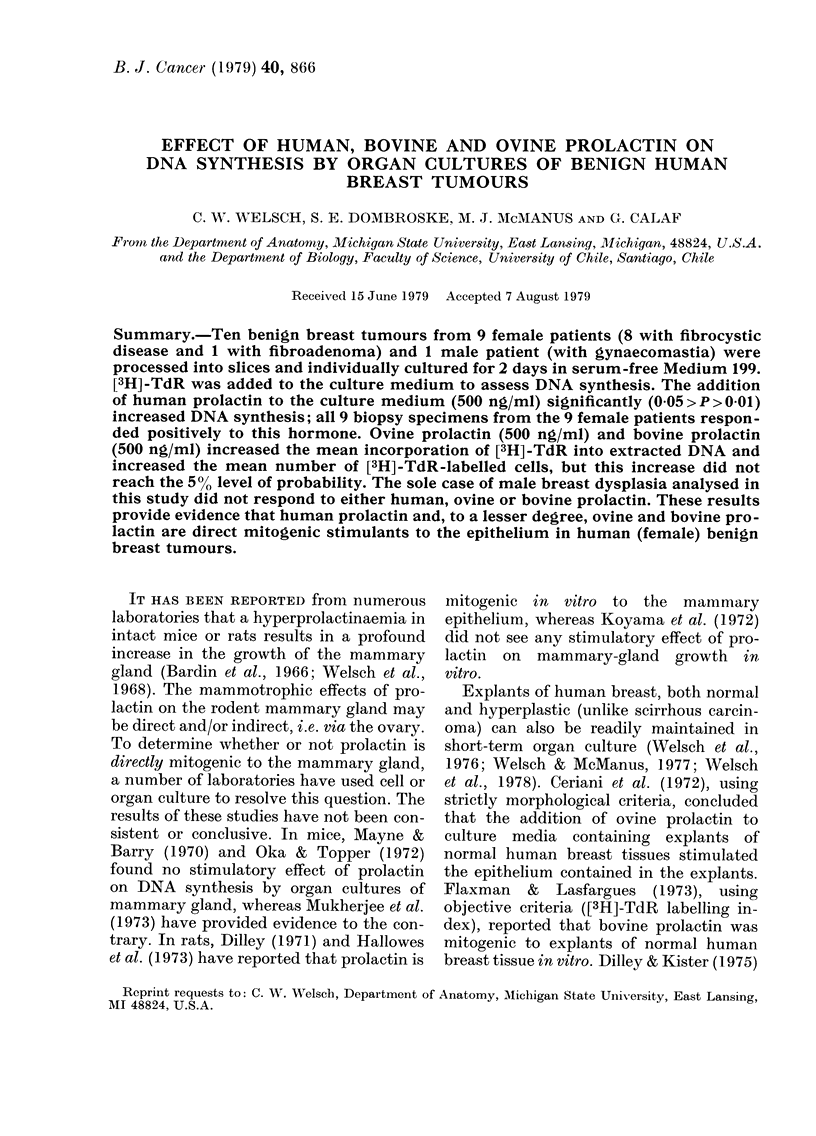

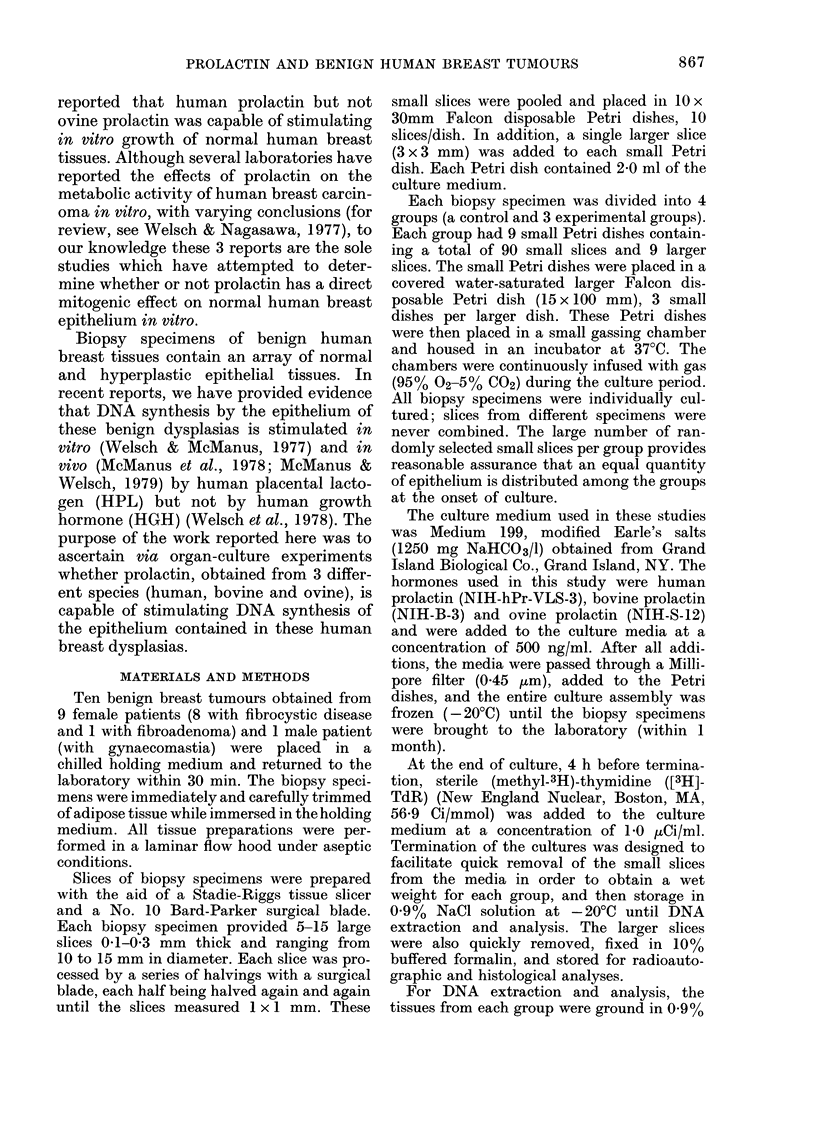

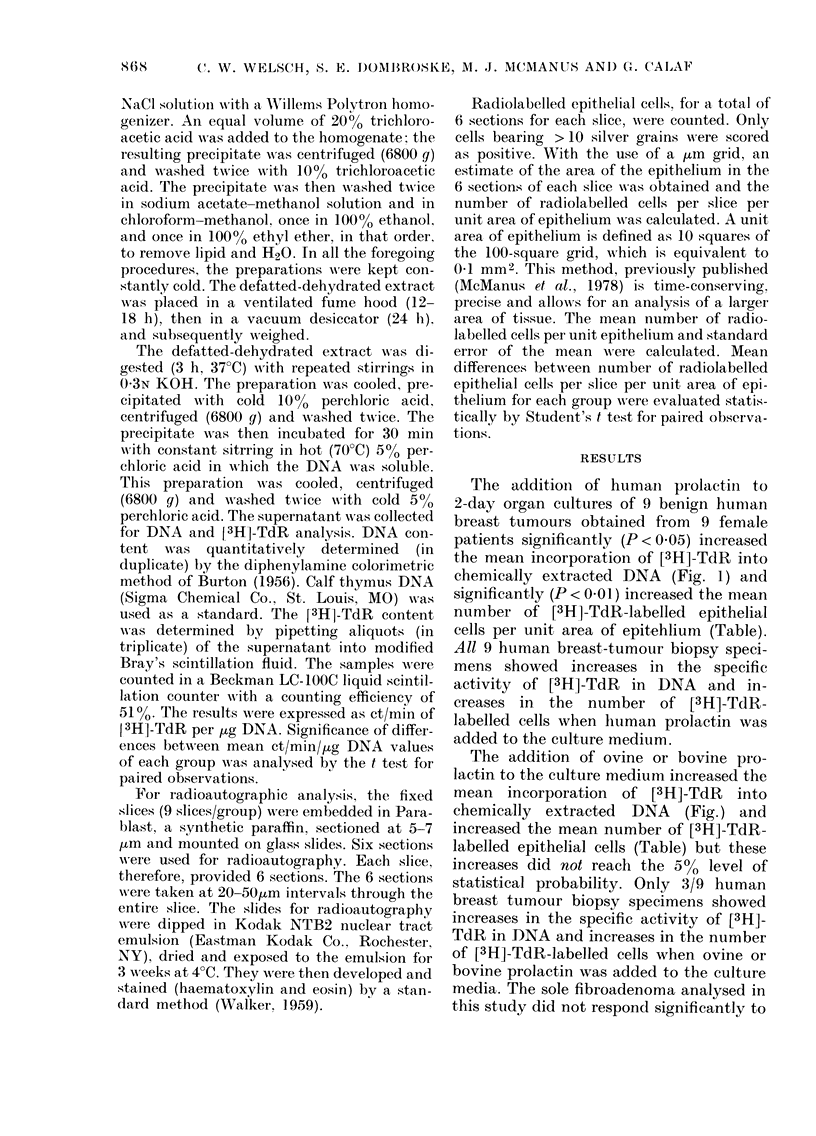

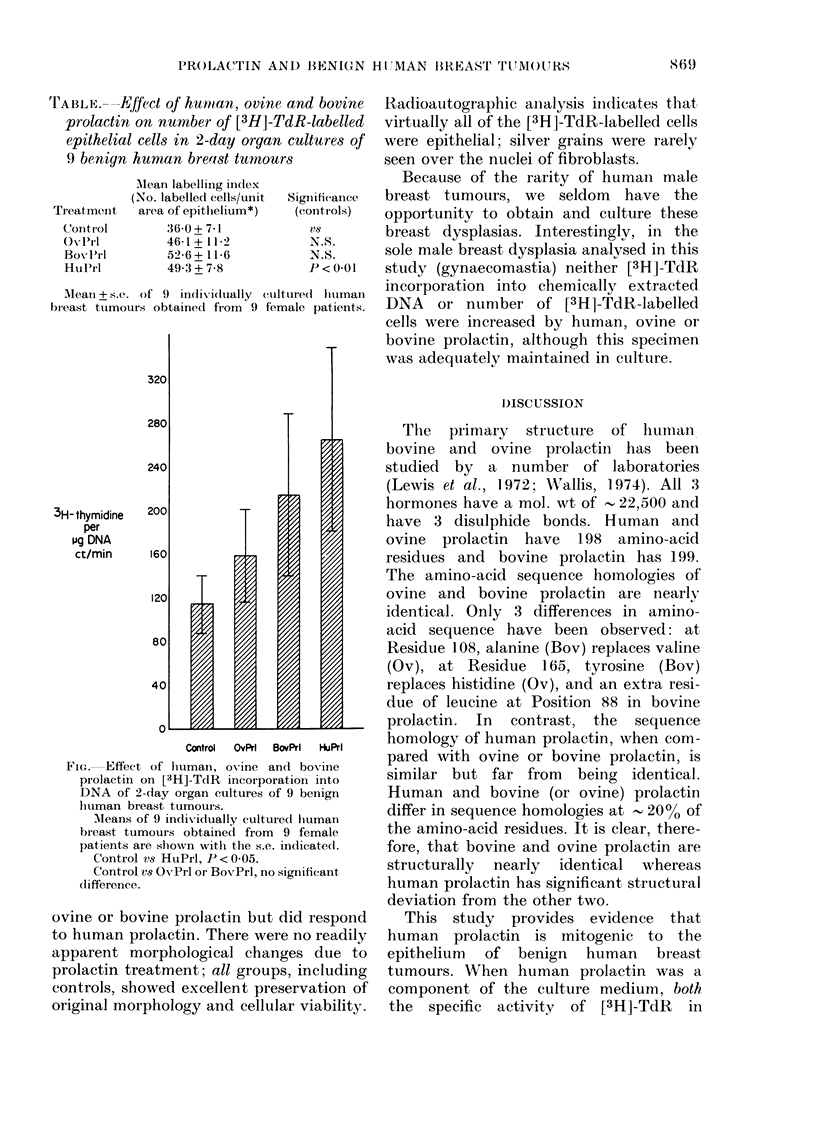

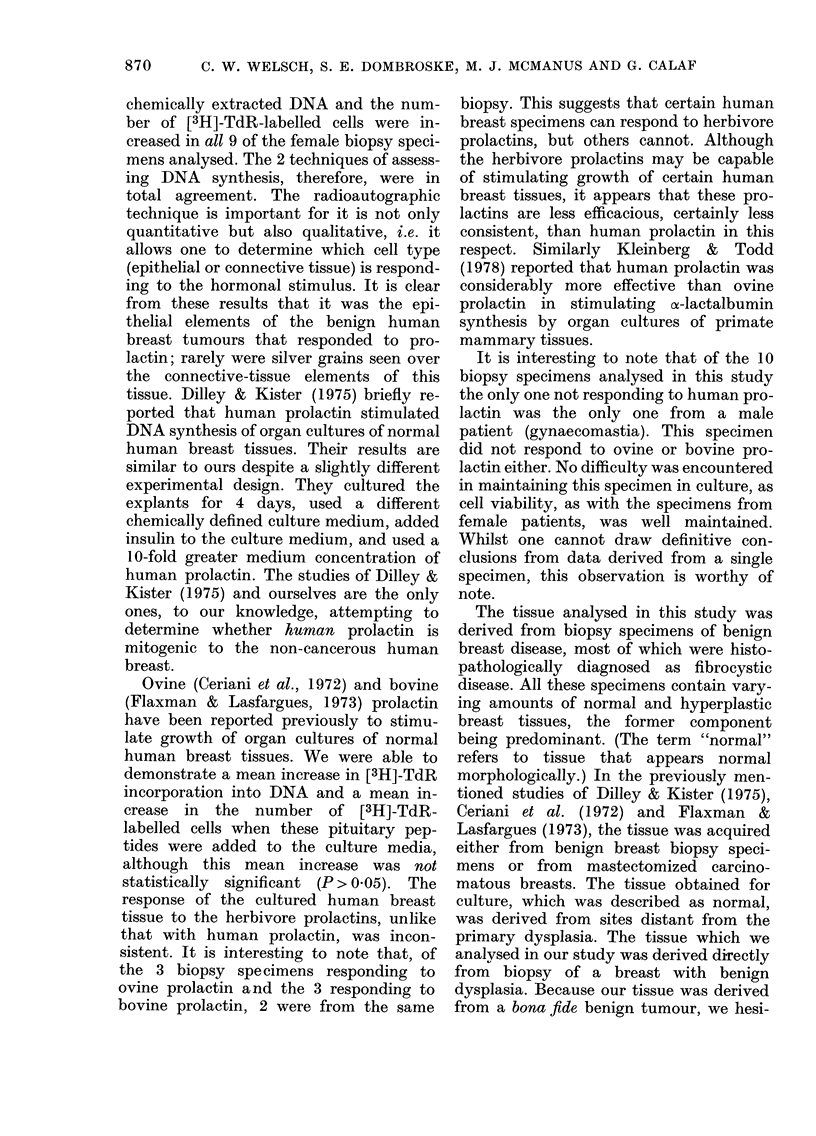

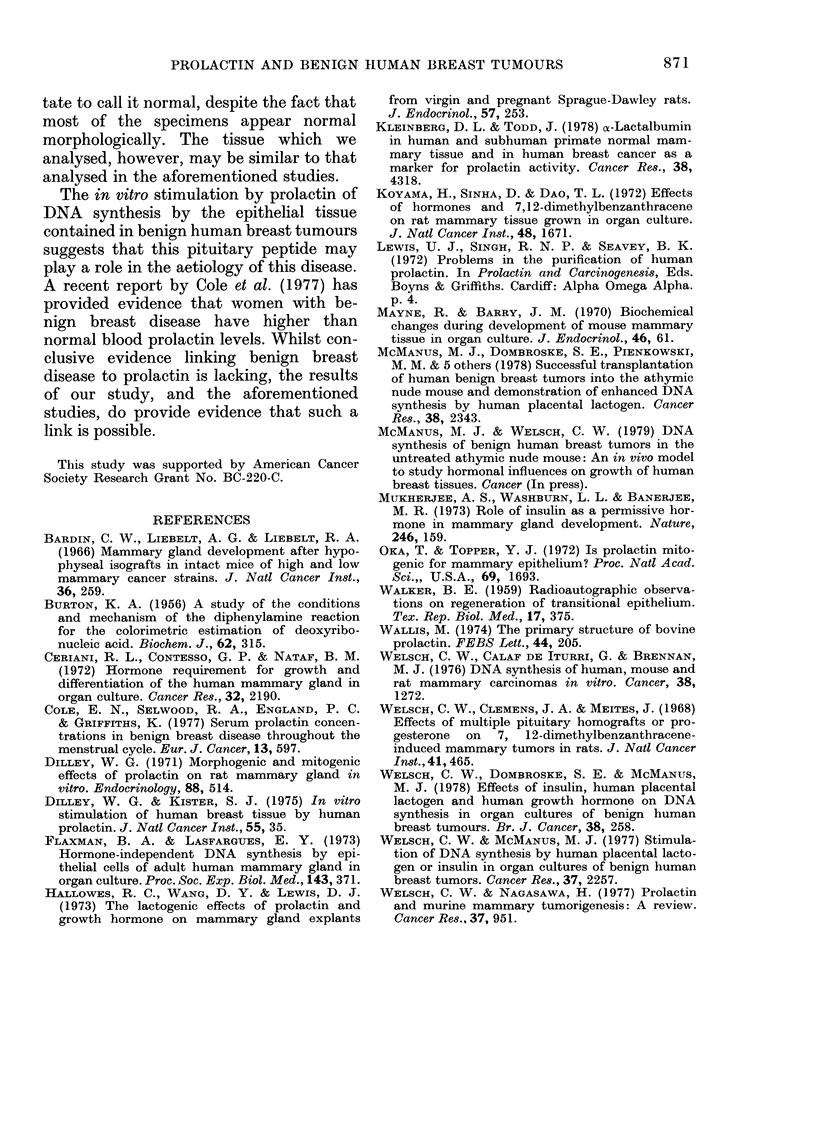


## References

[OCR_00610] BURTON K. (1956). A study of the conditions and mechanism of the diphenylamine reaction for the colorimetric estimation of deoxyribonucleic acid.. Biochem J.

[OCR_00603] Bardin C. W., Liebelt A. G., Liebelt R. A. (1966). Mammary gland development after hypophysial isografts in intact mice of high and low mammary cancer strains.. J Natl Cancer Inst.

[OCR_00616] Ceriani R. L., Contesso G. P., Nataf B. M. (1972). Hormone requirement for growth and differentiation of the human mammary gland in organ culture.. Cancer Res.

[OCR_00622] Cole E. N., Sellwood R. A., England P. C., Griffiths K. (1977). Serum prolactin concentrations in benign breast disease throughout the menstrual cycle.. Eur J Cancer.

[OCR_00628] Dilley W. G. (1971). Morphogenic and mitogenic effects of prolactin on rat mammary gland in vitro.. Endocrinology.

[OCR_00643] Hallowes R. C., Wang D. Y., Lewis D. J. (1973). The lactogenic effects of prolactin and growth hormone on mammary gland explants from virgin and pregnant Sprague-Dawley rats.. J Endocrinol.

[OCR_00651] Kleinberg D. L., Todd J. (1978). alpha-Lactalbumin in human and subhuman primate normal mammary tissue and in human breast cancer as a marker for prolactin activity.. Cancer Res.

[OCR_00658] Koyama H., Sinha D., Dao T. L. (1972). Effects of hormones and 7,12-dimethylbenz[a]anthracene on rat mammary tissue grown in organ culture.. J Natl Cancer Inst.

[OCR_00671] Mayne R., Barry J. M. (1970). Biochemical changes during development of mouse mammary tissue in organ culture.. J Endocrinol.

[OCR_00676] McManus M. J., Dembroske S. E., Pienkowski M. M., Anderson T. J., Mann L. C., Schuster J. S., Vollwiler L. L., Weisch C. U. (1978). Successful transplantation of human benign breast tumors into the athymic nude mouse and demonstration of enhanced DNA synthesis by human placental lactogen.. Cancer Res.

[OCR_00691] Mukherjee A. S., Washburn L. L., Banerjee M. R. (1973). Role of insulin as a "permissive" hormone in mammary gland development.. Nature.

[OCR_00697] Oka T., Topper Y. J. (1972). Is prolactin mitogenic for mammary epithelium?. Proc Natl Acad Sci U S A.

[OCR_00707] Wallis M. (1974). The primary structure of bovine prolactin.. FEBS Lett.

[OCR_00717] Welsch C. W., Clemens J. A., Meites J. (1968). Effects of multiple pituitary homografts or progesterone on 7,12-dimethylbenz[a]anthracene-induced mammary tumors in rats.. J Natl Cancer Inst.

[OCR_00724] Welsch C. W., Dombroske S. E., McManus M. J. (1978). Effects of insulin, human placental lactogen and human growth hormone of DNA synthesis in organ cultures of benign human breast tumours.. Br J Cancer.

[OCR_00711] Welsch C. W., Iturri G. C., Brennan M. J. (1976). DNA synthesis of human, mouse, and rat mammary carcinomas in vitro: influence of insulin and prolactin.. Cancer.

[OCR_00731] Welsch C. W., McManus M. J. (1977). Stimulation of DNA synthesis by human placental lactogen or insulin in organ cultures of benign human breast tumors.. Cancer Res.

